# Phenological Shifts in Wood Formation Tracked by Frost Rings Across Two Centuries

**DOI:** 10.1111/gcb.70745

**Published:** 2026-02-13

**Authors:** Eugenia Mantovani, Angela Luisa Prendin, Michele Brunetti, Davide Frigo, Raffaella Dibona, Marco Carrer

**Affiliations:** ^1^ Department of Land Environment Agriculture and Forestry (TeSAF) University of Padova Legnaro Italy; ^2^ Section for Ecoinformatics and Biodiversity, Department of Biology Aarhus University Aarhus Denmark; ^3^ Institute of Atmospheric Sciences and Climate National Research Council (CNR‐ISAC) Bologna Italy

**Keywords:** cambial phenology, climate change, European larch, *Larix decidua*, Norway spruce, *Picea abies*, *Pinus cembra*, Swiss stone pine

## Abstract

Accelerated warming, particularly in mountain regions, is altering plant phenology across ecosystems. However, the extent of these changes varies among species and regions. While phenophases in plant compartments such as leaves or flowers are relatively easy to observe, monitoring xylem phenology remains challenging due to the labour‐intensive methods required to capture intra‐annual growth dynamics. Here, we adopted an indirect retrospective approach to infer cambial phenology by analysing the timing and occurrence of frost damage in the growth rings of three Alpine conifer species. Increment cores were collected from 4481 individuals (1897 *Larix decidua* Mill., 980 *Picea abies* L. Karst., and 1604 *Pinus cembra* L.) at two high‐elevation sites in the Eastern Alps. Frost rings were identified, dated, and compared with long‐term (1774–2020) daily temperature records to determine their timing, using the 1.7°C threshold for cambial activity onset and subsequent episodes when minimum temperatures dropped below 0°C. We found that the cold spells responsible for frost ring formation remained consistent, typically involving temperatures dropping below freezing for an average of 4–5 consecutive days. However, the timing of frost ring formation, and thus cambial onset, has shifted over the past 200 years with no significant differences across taxa. This shift corresponds to about 7 days earlier per century or per °C of warming and is notably smaller than phenological shifts reported for other plant compartments in the same region. Given the critical role of cambial activity in forest carbon dynamics, these findings can help refine global vegetation models and improve predictions of ecosystem responses to climate change.

## Graphical abstract

Rapid warming is reshaping plant phenology across ecosystems. We present an indirect retrospective approach to infer cambial phenology by analysing the timing and occurrence of frost rings in three Alpine conifers. We found that the cold spells responsible for frost ring formation typically involve temperature dropping below freezing for an average of 4–5 consecutive days. However, the timing of frost ring formation, and thus cambial onset, has shifted to about seven days earlier per century or per °C of warming. This shift is notably smaller than the phenological changes reported for other plant compartments in the same region.

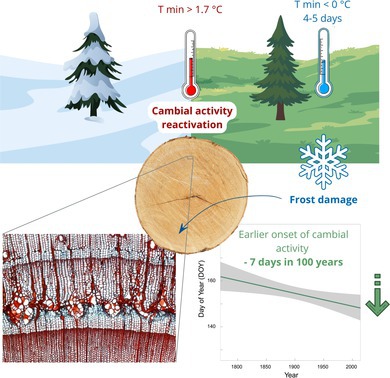

## 1 Introduction

Accelerated warming, especially at high latitudes and altitudes, driven by elevated greenhouse gas concentrations and concurrent feedback mechanisms within the climate system, is now evident (IPCC 2023). In mountain regions, warming rates appear to intensify with elevation, as temperature is influenced by a wide range of elevation‐specific climate factors, such as snow and ice cover, cloud patterns, water vapor, aerosols, and soil moisture, as well as positive feedback mechanisms, for example changes in surface albedo (IPCC 2022; Pepin et al. 2022; Rangwala and Miller 2012). This variability is further modulated by the complex topography of mountain systems, which leads to significant spatial and temporal heterogeneity in their climatic responses (Rangwala and Miller 2012), making mountain ecosystems particularly vulnerable to the impacts of rising temperature (I.‐C. Chen et al. 2011).

Most living organisms attempt to cope with this change by adjusting their phenology, a strategy that is often among the earliest observable responses to warming (Parmesan 2006). However, such changes in the timing of life cycle events, such as flowering, leaf‐out, and migration, can disrupt species interactions, influence resource availability, and alter competition dynamics within ecosystems (Inouye 2022; Vitasse et al. 2021). Across Europe, during the period 1959–1996, spring phenological events in plants advanced on average by 6.3 days, while autumn events were delayed by 4.5 days, resulting in an average lengthening of the growing season by 10.8 days (Menzel and Fabian 1999). These observations are in line with the earlier spring leaf onset of 2.2–2.5 days per decade reported by several investigations across the Northern Hemisphere and Europe (Menzel and Fabian 1999; Parmesan 2006; Schwartz et al. 2006; Zohner et al. 2020). In the European Alps, warming is occurring at a faster rate compared to the average across the Northern Hemisphere (Gobiet and Kotlarski 2020; IPCC 2022; Kotlarski et al. 2023; Rebetez and Reinhard 2008; Sommer et al. 2020). Here, due to the influence of rising temperatures and the shift in spring phenology, researchers also observed a concurrent uphill movement of the distribution ranges of numerous taxa, with significant changes in optimum elevation, particularly recorded for herbaceous and woody plants (Vitasse et al. 2021).

Unlike leaf phenology, which is relatively straightforward to study as it can be monitored using non‐invasive and direct techniques such as visual, phenocams or satellite remote sensing observations (Piao et al. 2019), xylem phenology is not directly observable and requires labour‐intensive methods to investigate, including microcore sampling at regular intervals, a specific laboratory processing procedure, and detailed anatomical and microscopic analyses (Cuny et al. 2015; Deslauriers et al. 2008; Rathgeber et al. 2016; Rossi et al. 2007; Silvestro et al. 2025). However, despite these challenges, studying xylem phenology remains crucial, as wood and forests constitute the largest carbon pool within the planet's living biomass. Xylem phenology not only shapes long‐term carbon accumulation, and thus the role of forests in the carbon cycle and climate change mitigation, but also provides key insights into how trees respond to environmental variability (Chen et al. 2022; Rossi et al. 2016). Cambial phenology is primarily influenced by temperature, which regulates the resumption of cambial activity necessary for wood formation (Rossi et al. 2014); although other environmental cues, such as photoperiod and winter chilling, can play a secondary yet appreciable role (Asse et al. 2018; Lin et al. 2024; Mu et al. 2023; Vitasse et al. 2018; Zhang et al. 2020). In heat‐limited environments, where growth is strongly constrained by temperature, warming trends may relax thermal constraints with faster and earlier reaching of the temperature sums required for growth onset in spring (Rossi et al. 2011). Extensive research on cambial phenology and xylogenesis has provided valuable insights into the intra‐annual dynamics of wood formation (Huang et al. 2020; Rossi et al. 2016). However, due to the challenges of acquiring yearly phenological information, most research works in this field hardly span more than a few years, posing a critical limitation to a thorough understanding of the multi‐year responses of cambium phenology to long‐term climate variability in long‐living organisms such as trees.

Frost rings are among the most commonly detected xylem anomalies and likely represent the ones with the longest tradition in tree‐ring studies (Harris 1934; Rhoads 1923). They represent distinctive features within a ring, characterized by cambial cells with damaged walls, deformed radial rays, and disrupted tracheids or vessels (Fritts 1976; Kaennel Dobbertin and Schweingruber 1995). These rings form as a result of environmental stress caused by unseasonal freezing temperatures during the growing season, which damages developing and still‐living cells, leading to the formation of characteristic anomalies (Glerum and Farrar 1966). From an anatomical perspective, below‐freezing temperatures and the potential formation of ice crystals in the cambial zone can disrupt normal cell wall development prior to lignification. This disruption may alter lignification processes, producing variations in cell wall thickness within the cellulose–hemicellulose–lignin matrix, and may also result in deformed or collapsed cells (Lee et al. 2007). Ultimately, such frost‐induced xylem injuries can compromise not only the wood mechanical properties but also its hydraulic efficiency, increasing the risk of conduits cavitation (Ma et al. 2024).

These anomalies appear in the earlywood or latewood, depending on whether the frost occurred after the typical winter dormancy season (i.e., late frost events) or at the end of the vegetative period (i.e., early frost events). Frost rings provide clear and precise markers and have been used to reconstruct past climate conditions, particularly extreme cold spells, even associated to the global impact of significant volcanic eruptions (D'Arrigo et al. 2001; LaMarche and Hirschboeck 1984; Salzer and Hughes 2010), species susceptibility, and adaptation (Payette et al. 2010). Although the warming trend generally reduces the number of frost events per year, it could also potentially extends the growing season in temperature‐limited ecosystems (Liu et al. 2018). In this way, vegetation can face an extended period during which photosynthetically active species are exposed to frost (Liu et al. 2018). Recent findings show that spring frost days have increased, particularly in Europe, over the last three decades, indicating greater vegetation exposure to cold spells (Liu et al. 2018).

Here, we used frost rings as proxies to retrospectively investigate the cambial phenology of three key conifer species growing across the Alps: European larch ( *Larix decidua* Mill., LADE), Swiss stone pine ( *Pinus cembra* L., PICE), and Norway spruce ( *Picea abies* L. Karst., PIAB). Since frost rings typically occur at the very beginning or end of the growing season, their presence provides direct evidence that the cambium had already been reactivated or was still active when the cold spell occurred. To reconstruct cambial phenology and quantitatively identify the climatic conditions that promote frost ring formation, we combined frost ring occurrence data with detailed daily temperature records spanning two and a half centuries. Furthermore, we aimed to evaluate the impact of 20th‐century warming on cambial activity and assess whether this aligns with phenological trends observed in other vegetative compartments, such as leaf and flower onset.

## 2 Material and Methods

### 2.1 Study Site

**FIGURE 1:**
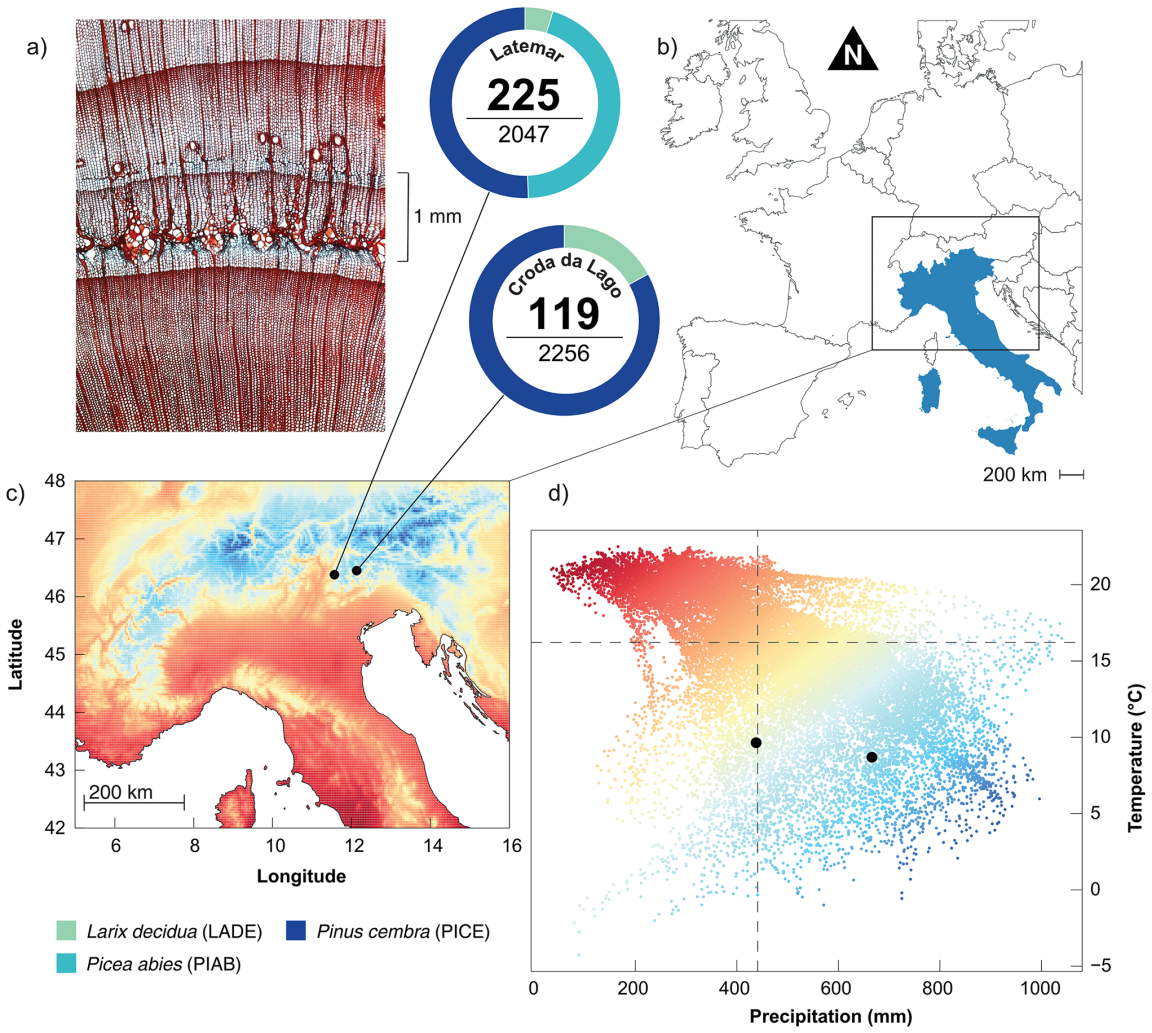
(a) Anatomical section showing a frost ring of *Pinus cembra* L. (b) Map of the broader study area (black polygon). (c) Locations of the two sampling sites (black dots) Latemar (left) and Croda da Lago (right), with the number of series containing frost rings relative to the total number of series. (d) position of study sites within the climatic space. Colour gradients represent the interaction between the two climate variables (May to September mean temperature and total precipitation), ranging from red (warm, dry) to blue (cold, wet). Perpendicular dashed lines indicate the median values of each variable. Map lines delineate study areas and do not necessarily depict accepted national boundaries.

The study sites coincide with two 4‐ha permanent plots, Croda da Lago (CRO, 46.45 N, 12.13 E) and Latemar (LAT, 46.23 N, 11.32 E), established in the 1990s in the Eastern Italian Alps (Figure 1). Both share the same settings with dolomite and limestone bedrock, rendzic leptosols soils (Carrer and Urbinati 2004), North‐West/North‐East aspect and an elevation between 1900 and 2100 m above sea level. Both stands feature mixed subalpine forests composed of European larch ( *Larix decidua* Mill.), Swiss stone pine ( *Pinus cembra* L.), and Norway spruce ( *Picea abies* L. Karst.) that have not been managed or affected by major disturbances for decades (Carrer et al. 2018; Carrer and Urbinati 2001). The climate in this area of the Dolomites region is characterized by dry winters, with most of the precipitation occurring during summer and autumn. Annual precipitations are usually over 1000 mm, with the CRO site being slightly wetter. In both areas, the coldest and hottest months are January and July, respectively, with the LAT site being on average 1°C warmer Figure S1.

### 2.2 Sample Collection and Preparation

All standing trees within the permanent plots with a diameter at breast height greater than 8 cm were sampled. From each tree, one increment core was extracted at 1.30 m above ground (breast height), oriented perpendicular to the slope. A total of 4481 trees were sampled, 2434 at the CRO (1579 larches, 677 Swiss stone pines and 178 spruces) and 2047 at LAT (318 larches, 927 pines, and 802 spruces).

At the laboratory, all cores were mounted on wooden boards and polished with progressively finer grain sandpaper to obtain a clear visualization of the growth rings. The ring width was measured to the nearest 0.01 mm then each ring‐width series was checked, corrected, and crossdated, both visually and statistically, using COFECHA (Holmes 1983). During measuring and then with a second thorough inspection performed after crossdating, special attention was given to visually identifying and dating the occurrence of frost rings within each core.

### 2.3 Climatic Data

We reconstructed daily climatic information specific to the sampling sites for the past 250 years by utilizing the anomaly method (Mitchell and Jones 2005) and the extensive instrumental data available for the Alpine region dating back to the late eighteenth century.

The anomaly method is a widely used technique for interpolating station series to a specific geographic point, ensuring that variations in data availability across stations do not bias the results. This method involves first converting the temporal series into anomalies relative to a common reference period. These anomalies are then interpolated for the specific site, after which the mean climate values representative of the site are added to the interpolated series, converting the anomalies back into absolute values. Specifically, the approach allows to reconstruct monthly climatologies (i.e., mean climate values over a specific reference period) and their relative deviations (i.e., anomalies with respect to the same baseline period). Climatologies, exhibiting strong spatial gradients, are reconstructed using the many weather stations available, even if their records cover only short periods, combined with interpolation techniques that account for the dependence of climatic variables on orography (Brunetti et al. 2014). In contrast, anomalies linked to climate change and variability exhibit higher spatial coherence. Therefore, a smaller number of stations with satisfactory temporal coverage can effectively capture spatial patterns using simpler interpolation techniques, although data homogenization is essential. Homogenization ensures corrections for errors arising from the historical context of the stations, such as changes in station location, instrument replacements, and similar factors.

Monthly minimum and maximum temperature series were derived by superimposing the reconstructed climatologies and anomalies (Brunetti et al. 2012). After reconstructing the monthly series for each site, the methodology of Di Luzio et al. (2008) was applied to obtain daily values. Specifically, the daily values of each station series of the reference dataset were converted into relative contributions to the total monthly amounts and spatially interpolated to the same sites as the monthly data. The previously reconstructed monthly series were then used as constraints to convert the relative contributions back into absolute daily amounts. Data were reconstructed for the period 1774–2020 adopting a maximum searching radius of 350 km from each site.

The base of data used for the interpolation results from years of data rescue and homogenization activities, using core datasets presented by Brunetti et al. (2006) for Italy and by Auer et al. (2007) for the HISTALP project. Over the past two decades, most of these climate records have been recovered at a daily resolution, and the dataset has been extended with hundreds of additional station records obtained through numerous data rescue initiatives conducted by various Italian regional environmental agencies. These series were subsequently subjected to the same homogenization procedure, as detailed in (Brunetti et al. 2006) and Simolo et al. (2011), with performance assessments described in Venema et al. (2012).

To assess the performance of the interpolation technique at each site, the full interpolation procedure was validated using a leave‐one‐out approach. This validation was applied to the daily minimum and maximum temperature series of the last 25 years from stations within a 25 km pooling distance of each site (39 stations for minimum and 41 for maximum temperature at LAT; 48 and 50 for minimum and maximum temperature, respectively, at CRO). Specifically, each station's temperature series was independently reconstructed by excluding the station itself and using only the remaining observations. The reconstructed and observed daily series were then compared to evaluate model performance. To further evaluate how reconstruction performance varied through time in relation to changes in station availability, we assessed the reconstruction by splitting the full time interval into three sub‐periods of equal length (1774–1855, 1856–1937, and 1938–2020) and computing the same verification statistics as described above over the last 25‐year period, considering as references for the reconstruction only the stations available in each sub‐period. Correlation coefficients (r) between observed and modelled series were consistently high, exceeding 0.90 for both minimum and maximum temperature at both sites across all the three subperiods. After removing the dominant annual cycle to specifically assess day‐to‐day variability, correlations decreased slightly, with only one value falling below 0.8 (0.79 for minimum temperature at Croda in the earliest period); all correlations were significant at *p* < 0.001. The full set of correlation coefficients, together with additional error metrics (BIAS, MAE and RMSE), confirmed the high and stable reconstruction skill of the interpolation method (Table S1). Importantly, the reconstruction demonstrates a particularly strong capacity to capture cold spells and to correctly identify frost‐day timing (Figure S2). This is critical given that the primary objective of this study was not the reconstruction of absolute temperature values, but rather the accurate detection of the timing (day of the year) of frost‐damage occurrence.

### 2.4 Detection of the Frost Event Inducing Cambial Injury

According to previous investigations in the same area and on the same species, Rossi et al. (2007) identified the following temperature ranges as necessary to support the cellular processes involved in wood formation: minimum temperatures 1.7°C–4.8°C, mean temperatures 5.6°C–8.5°C, and maximum temperatures 10.9°C–13.3°C. Using these reference thresholds together with the reconstructed site‐specific daily temperature records, we determined, for each calendar year in which at least one frost ring was observed, the Julian day when cambial activity likely began. We then moved along the daily temperature records to assign the specific day of frost ring formation, defined as the day when the lowest minimum temperature below 0°C was reached, likely disrupting the normal process of wood formation due to freezing stress.

### 2.5 Statistical Analysis

For all sites together (Regional), for species and sites separately, as well as for each combination of species and site, we fitted linear, power, and exponential functions to identify which function best described the temporal trend in frost ring formation (Table S2). The representative models were chosen based on the explained variance and Akaike's Information Criterion (AICc) using the maximum‐likelihood method. Then, we assessed the role of frost event frequency in relation to both the day of the year (DOY) occurrence and the calendar year at the two sites and for the three species. We used Linear Mixed Effects Models (LMM) when analysing multiple sites and species together, and linear models (LM) when examining individual sites and species or testing differences among them (Table S3). The model fits were evaluated by the residual and fitted values (Zuur et al. 2009). The LMM accounted for the day‐year associations within sites as well as the occurrence variability among years (Gazol and Camarero 2012). The DOY with the lowest minimum temperature below zero following the onset of the growing season, and thus likely causing the frost ring, was considered as a response variable. The calendar year (Year), tree age at the moment of frost occurrence (or mean age when multiple trees within each species–site combination were present), and the relative frequency of frost rings, calculated as the number of frost rings per year divided by the number of sampled trees of each species and site (Frequency), were included as fixed effects. To account for possible temporal autocorrelation we also included AR(1) in the initial models. Species‐Site was considered a random effect when analysing multiple sites and species to account for group‐level variability (Crawley 2007). The representative models were chosen based on the explained variance and Akaike's Information Criterion (AICc) using the maximum‐likelihood method (Zuur et al. 2009).

To analyse the timing of frost events, we used linear mixed‐effects models (LMMs) that accounted for both fixed effects and the hierarchical structure of our data. The final model can be written as:

(1) $${\text{Days}}_{i}=\beta_{0}+\beta_{1}\cdot {\text{Year}}_{\mathrm{ij}}+\beta_{2}\cdot {\text{Frequency}}_{j}+\text{Species}\_\text{Site}+\varepsilon$$

where *β*_*0*_ is the intercept, *β*_*1*_ and *β*_*2*_ are fixed‐effect coefficients. The term Species_Site is a random intercept that allows each species‐site combination to have its own baseline timing of frost events, capturing group‐specific variation and the non‐independence of observations within groups. The residual term ε allows for variation in the intercept of Year across species‐site combinations. To account for potential temporal autocorrelation, we also tested the model including an AR(1) correlation structure. However, the inclusion of this structure did not improve model fit (Figure S3, Table S4).

Finally, we also use linear models to assess the intra‐ and inter‐specific variability of the occurrence of frost events over the last couple of centuries in each site and for each species and their combinations using the following model:

(2) $${\text{Days}}_{i}=\beta_{0}+\beta_{1}\cdot {\text{Year}}_{\mathrm{ij}}+\beta_{2}\times \text{Species}\_\text{Site}+\beta_{3}\cdot \left({\text{Yeari}}_{j}\cdot \text{Species}\_\text{Site}\right)+\varepsilon$$

In this model, *β*_*0*_ is the intercept, *β*_*1*_ is the main effect of calendar year, *β*_*2*_ represents differences among species‐site combinations, and *β*_*3*_ captures interactions between *Year* and each group. This model enables us to assess whether temporal trends in frost ring timing differ among species and sites.

Pairwise comparisons of slopes were conducted using the *emtrends* function from the *emmeans* package to assess significant differences. We accounted for assumptions of normality and homoscedasticity, verifying the normal distribution and random distribution of residuals in all models (Zar 1999; Zuur et al. 2009). Multicollinearity was assessed using variance inflation factors (VIF) (Table S5). The ‘lme4’ package in R was used to perform the analyses, and the significance of the fixed effects was tested with F‐tests (Bates et al. 2015; Pinheiro and Bates 2000) while the variance explained by the fixed and random effects (R^2^ conditional) was calculated for each model using the lmerTest package (Kuznetsova et al. 2017).

This approach allowed us to thoroughly assess the overall temporal trends in frost timing while accounting for variation among species and sites. The inclusion of random intercepts and interactions ensured that group‐specific deviations were accounted for, providing a robust framework for analyzing long‐term patterns in frost occurrence.

To assess the stability of our results and, in particular, how tree age structure and the Julian day setting of the frost event could influence the derived phenological trends, we performed a multiple bootstrap sensitivity analysis (100,000 replications). Specifically, we tested (i) age restrictions, excluding trees younger than 5 and trees younger than 10 years at breast height at the time of frost injury occurrence; (ii) sequential exclusion of trees in successive 10‐year age classes; (iii) stratified resampling within each 10‐year age class, proportional to its distribution in the reference year, thereby preserving the original age structure; (iv) an unbalanced inverse‐probability weighting resampling scheme to correct for unequal representation among age classes, assigning greater weight to underrepresented classes; and (v) applying a random temporal offset of ±2 and ±3 days in the Julian day identification of frost events (Figure S4 and Table S4). Moreover, we tested the effect of tree age by including it in the LMMs (Table S6).

To provide additional insights into the climatic conditions determining frost ring formation, we applied Superposed epoch analysis (SEA) (Panofsky and Brier 1958) to identify and quantify temperature dynamics before and after frost events. The SEA was centred on the day with the lowest minimum temperature during the cold spell, enabling us to capture the full magnitude of the temperature anomaly associated with frost rings and ensuring consistency with our definition of a frost day. The same SEA was applied at the individual ring‐width series to test for potential legacy effects of frost damage at both the site and species–site group levels (Figure S5). In this case, all series were previously standardized using a 20‐year cubic smoothing spline, an effective method for isolating the climate signal by filtering out age‐related trends and potential disturbance pulses in these species and region (Carrer and Urbinati 2004; Cook and Peters 1981). The procedure involved fitting the spline function to each individual ring‐width series and calculating the ratio of observed to expected values, yielding dimensionless series subsequently used in the SEA.

Statistical analyses and visualisations were conducted using the software R (version 4.1.3).

## 3 Results

Frost rings were found exclusively in the first half of the growth rings, suggesting that at both sites, they were solely caused by late frost events occurring early in the growing season.

**FIGURE 2:**
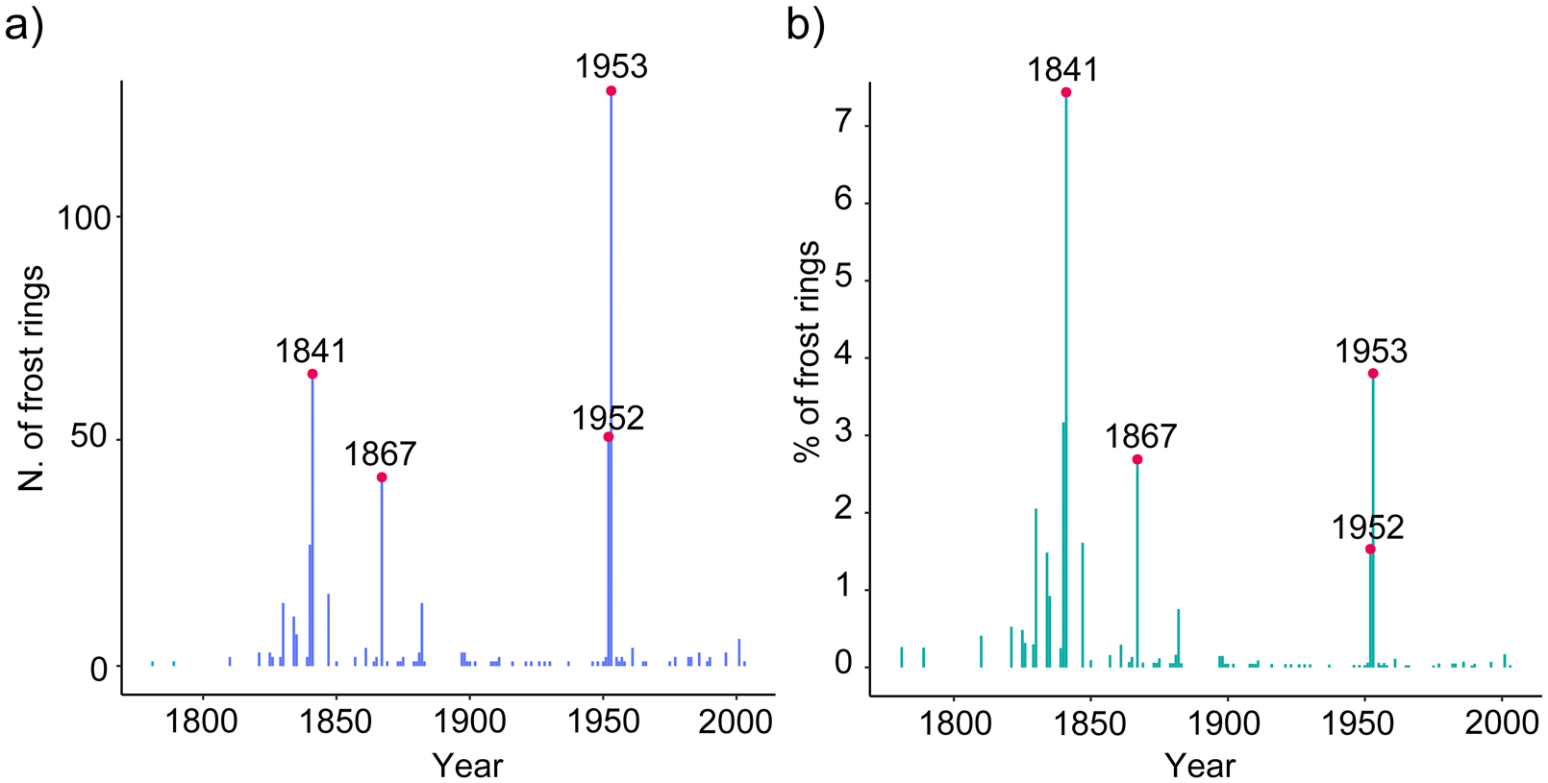
(a) Absolute number of frost rings over time and (b) percentage of frost rings expressed as proportion of trees that had a growth ring in that specific year. Red dots and labels highlight the years with the highest occurrence of frost rings.

The years with the highest incidence of frost rings were 1841, 1867, 1952, and 1953 (Figure 2). However, when considering the rate of frost rings relative to the number of sampled trees, 1841 stands out as the most significant year. During June 1841, both study sites experienced a severe frost spell that began on June 6 and lasted for at least 8 days, with temperatures dropping to −3.9°C. In contrast, 1953 showed the highest absolute occurrence of frost rings, particularly at the CRO site. This year was marked by a severe frost event lasting 9 days from late May into early June, with temperatures reaching as low as −7.1°C.

Frost events occurred between the 120th and 200th day of the year (DOY), from late April until mid‐July, with species‐specific and site‐specific differences.

**FIGURE 3:**
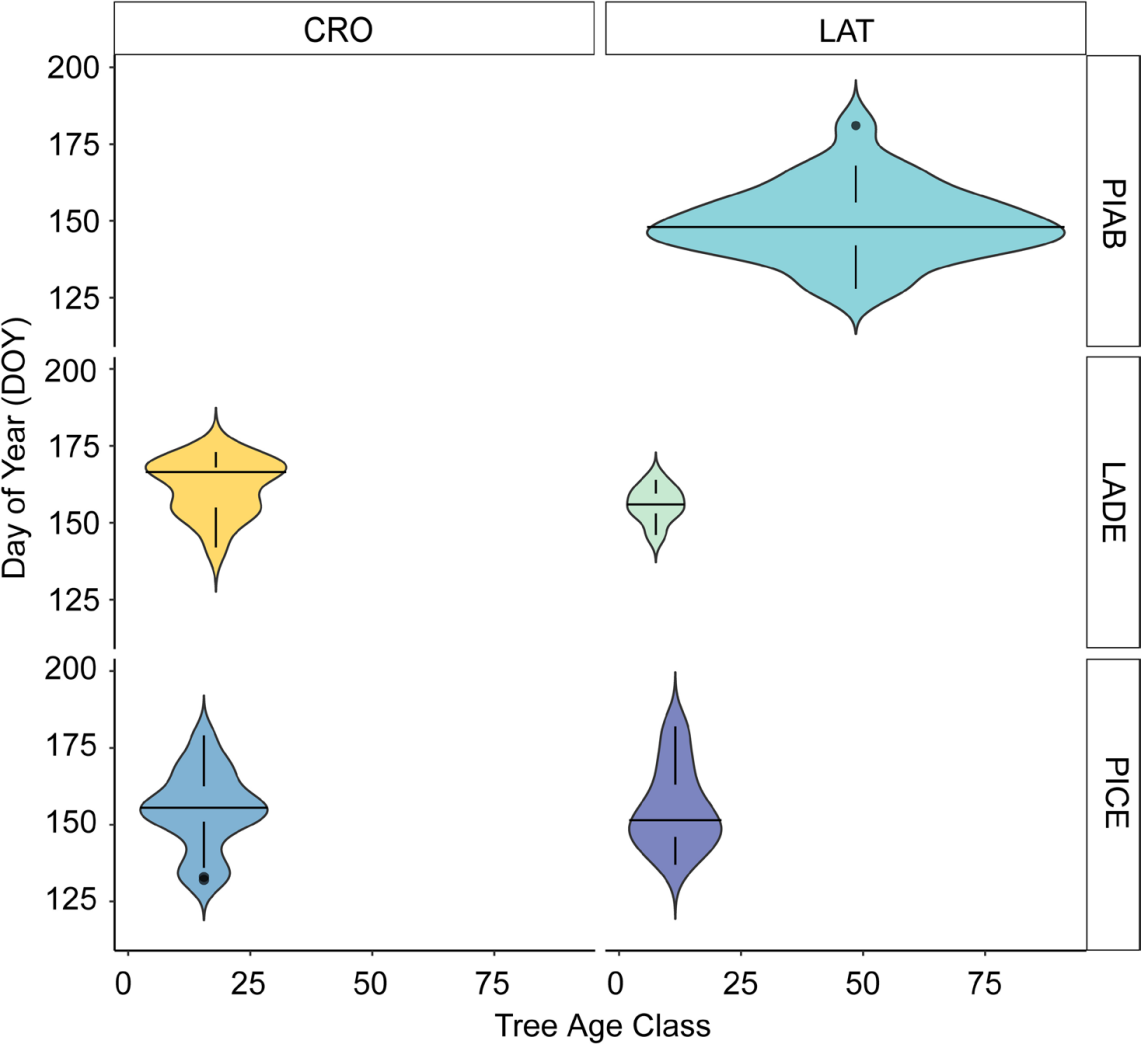
Distribution of the timing of frost ring formation (Day of Year, DOY) across different species and sites. The x‐axis represents tree age at the moment of frost ring formation, while the y‐axis indicates the DOY of frost ring formation. The violin plots display the density of the data, horizontal line represents the median, and whiskers extending to 1.5 times the interquartile range. Points outside this range are outliers.

The age of trees at the time of frost ring formation is generally similar across groups (Figure 3). Most groups exhibit a relatively narrow age range, with frost rings predominantly forming in trees younger than 25 years at breast height. Larch and Swiss stone pine show few, if any, occurrences of frost rings in older individuals. In contrast, Norway spruce displays a broader age distribution, with frost rings observed in trees up to approximately 80 years old.

**FIGURE 4:**
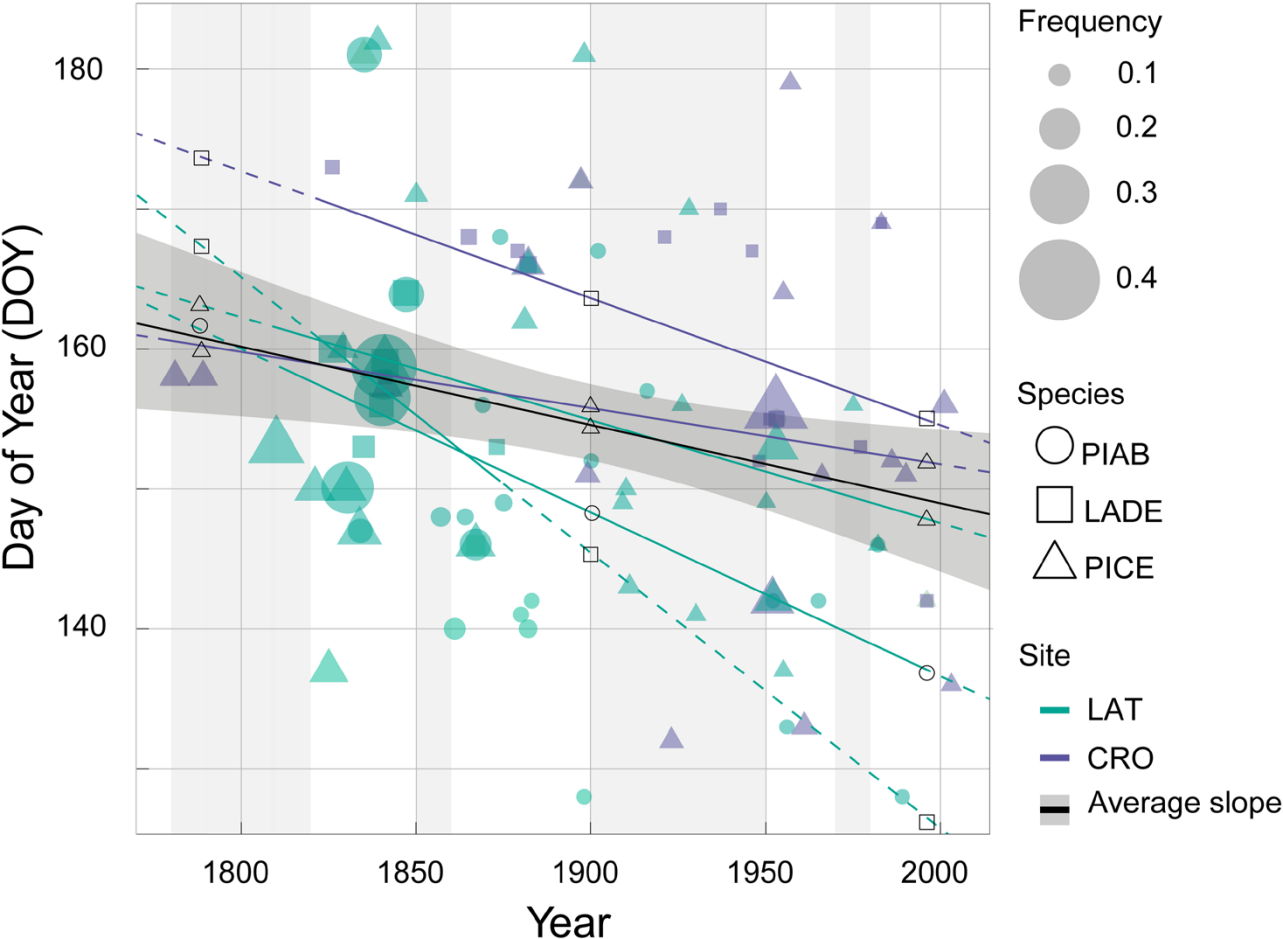
Day of the year (DOY) where frost rings have been detected. Point size indicates the frequency of frost rings occurrence and the linear regression lines show the relationship between DOY and the Calendar Year for each species and site. Grey vertical shading highlights decades with fewer than five frost‐ring events per decade.

The timing of frost events appears to have shifted earlier in the growing season across all species–site combinations, as evidenced by the significantly negative relationship between DOY and Year (Figure 4). Despite minor variations in slope magnitude among sites and species, the linear model did not reveal any statistically significant differences in the rate of change across species–site groups, indicating a broadly consistent temporal trend.

The LMM model used to assess the temporal shift in the timing of frost ring formation and the influence of frost ring frequency showed that the effect of Year was statistically significant (estimate = −0.070 ± 0.023 days year^−1^, *p* = 0.003), corresponding to an average advance in frost rings formation of approximately 7 days per century. In contrast, *Frequency (p = 0.977)* and *Age (p = 0.449)* were not significant predictors of timing in both models that considered or not the autocorrelation, indicating that neither the intensity of frost occurrence within a site‐year nor the age of the tree at the time of the frost event affected the timing of frost ring formation. The bootstrap sensitivity analyses with age restrictions and stratified resampling further confirmed that the tree age structure did not influence the observed phenological trends (β±95% percentile confidence intervals < 0, Figure S4). Results were also confirmed robust when considering possible bias in the setting of the Julian day of frost occurrence. Parametric bootstrapping showed that the Year effect remained negative and significant (β±95% percentile confidence intervals: −0.069 ± 0.044 and −0.067 ± 0.041 for Days±2 and Days±3, respectively), confirming a consistent long‐term temporal signal (Figure S4).

Post hoc comparisons of slopes, using Tukey's adjustment for unequal sample sizes, confirmed that the rate of cambial phenology advance did not significantly differ between species‐site groups (all adjusted *p*‐values > 0.7).

### 3.1 Superposed Epoch Analysis

**FIGURE 5:**
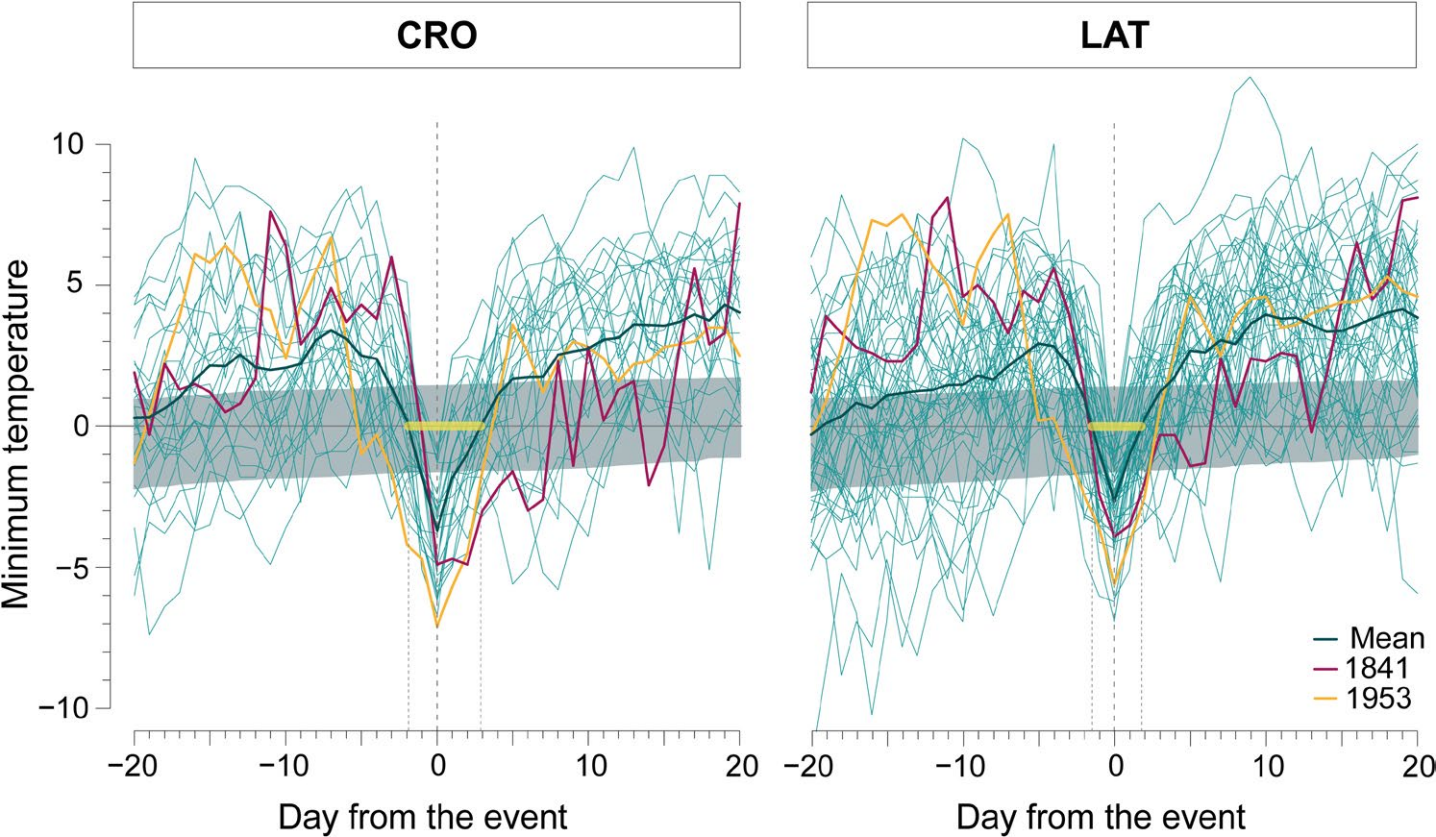
Superposed Epoch Analysis (SEA) of daily minimum temperature before and after frost‐ring formation events at the two study sites. Thin lines represent the daily minimum temperature over a 40 days period (20 days before and 20 after the first frost day) for each year in which we detected a frost ring. The bold green line represents the mean temperature across all the frost‐event years, while the horizontal yellow segment indicates the average duration of the cold spell. Red and orange lines highlight the years with the highest absolute (1953) and relative (1841) number of frost rings across both sites. The shaded grey area indicates the confidence interval. Day 0 (dashed vertical line) marks the day with the lowest temperature below 0°C during the cold spell.

The SEA unveiled the progression pattern in the minimum temperature around frost events (Figure 5). Approaching the first day of sub‐zero minimum temperature after the onset of the cambial activity, temperature rapidly drops, reaching the minimum value on the day of the event (Day 0). In the following days after the frost event, temperature progressively increases. The average tendency, represented by the bold line, shows a statistically significant initial and consistent drop in temperature around the cold spell, followed by a recovery phase. During the event, the minimum temperature reached an average of around −1.5°C/−2°C, a peak minimum of −3.7°C at CRO and −2.8°C at LAT and remained below 0°C for an average of 4 days (5 at CRO and 3 at LAT). SEA, applied on yearly ring‐width series, detected no significant legacy effects on tree‐ring growth up to 5 years after the frost damage (Figure S5).

## 4 Discussion

This study aimed at assessing the impact of climate warming on tree cambial phenology and to determine whether it synchronises with the advancing trends observed in the phenological onset of other vegetative compartments, such as leaves and flowers. We adopted an indirect retrospective approach to reconstruct cambial activity by analysing the occurrence of frost rings in conjunction with detailed daily temperature records spanning nearly two centuries.

The formation of frost rings is clearly associated with minimum temperatures of 0°C or below during the growing season and susceptibility varies depending on several factors, including tree age, species and the microtopographic environmental settings. Nonetheless, the temperature threshold for frost ring formation in conifers under natural conditions remains debated. Glerum and Farrar (1966) established that to obtain frost rings in the wood of various species of conifer trees, a temperature around −8°C. Other studies show that frost rings in black spruce become important when night temperatures fall below 0°C for 6 h, reaching a climax of −5.7°C (Dy and Payette 2007), or even by −5°C for 2 h (Nilov and Chertovskoi 1975). However, many of these previous studies on frost ring formation were performed in controlled experimental conditions on very young and small seedlings or saplings. Through a retrospective approach based on daily minimum temperature rather than hourly minima, our study shows that, in the field and with adult trees, the formation of a frost ring requires the persistence of sub‐zero temperatures for at least 2 days, with an average of 4 days, reaching an average minimum temperature of −2°C. This duration reflects the persistence of cold conditions and underscores the temporal span of frost events in the analysed dataset.

Our samples contained frost rings caused exclusively by late frost, one of the most critical extreme climatic events in temperate and boreal regions (Liu et al. 2018; Zohner et al. 2020). Recent studies have reported a significant increase in leaf‐damaging spring frost events, particularly in Europe and East Asia (Zohner et al. 2020). Our data confirmed that, across the Alps, the risk of cambial damages due to frost is restricted to the early part of the season, with no sign of frost injuries occurring late in the season over more than two centuries of records. The age distribution of frost‐ring formation at our sites reveals that younger trees are disproportionately affected, suggesting higher cambial vulnerability to frost during early developmental stages. Two main processes likely underlie this age‐related divergence: (i) xylogenesis exhibits an age‐related onset, with older trees typically starting cambial activity 2–3 weeks later than younger ones (Li et al. 2013; Rossi et al. 2008), potentially allowing them to avoid late frost damage and (ii) young trees have thinner bark (Glerum and Farrar 1966; LaMarche and Hirschboeck 1984; Payette et al. 2010). As trees mature, bark thickness increases, providing greater insulation against frost events and leading to a marked decline in frost‐ring frequency. This pattern is particularly evident in larch, a species characterized by pronounced age‐related changes in bark thickness, where frost‐ring frequency decreases from juvenile to mature wood (Payette et al. 2010), as observed in the individuals analysed in our study.

The gradual advancement of phenological events following the increase in temperature likely reflects the dynamics of spring warming across ecosystems (Silvestro et al. 2025). Leaf spring phenology is strongly influenced by temperature, and its earlier onset in recent years is widely recognized as clear evidence of climate change affecting Earth's vegetation (Vitasse et al. 2022). Fu et al. (2014) reported an advancing trend in leaf unfolding by 4.2 days per decade for six deciduous tree species in Europe. This advance outpaces the slower adjustments observed in xylem phenology, such as the onset of cell enlargement and the start of secondary cell wall formation. However, since leaf and xylem phenology are not linearly related and respond to different environmental controls, shifts in leaf phenology may not directly influence intra‐annual radial growth (Rossi et al. 2009). Radial growth mainly depends on the duration and rate of cambial activity and cell enlargement. Our results indicate an overall 14‐day advancement in frost ring formation over the past 200 years, suggesting an earlier onset of cambial activity. Specifically, the onset has shifted from the first half of June in the early 1800s to the last week of May in recent decades. A model by Rossi et al. (2011), estimated for *Picea mariana* [Mill.] B.S.P. (black spruce) in Canada, suggests that a 1°C increase in average annual temperature lengthens the total period of xylem growth from 8 to 11 days. It also indicates an earlier onset of cambial activity by approximately 4–5 days °C ^−1^, a slower pace compared to leaf phenology shifts. In the same region and for the same species, Boulouf Lugo et al. (2012) estimated an earlier onset of 0.5–0.8 days/decade from 1950 to 2010. Our results emphasize that, despite differences in latitude and taxa, the warming trend recorded (+2°C between 1800 and 2005) corresponds with an earlier onset of cambial phenology. Specifically, we estimate a shift of approximately 7.2 (± 2.4) days per °C, roughly in between what was predicted for black spruce in Canada in the two abovementioned studies. This value is also consistent with the results of Moser et al. (2010) for larch in the Swiss Alps, who reported a growing season lengthening of 3–4 days per 100 m in elevation, corresponding to 7 days °C^−1^. Though some inconsistencies may also stem from the different time frames of the investigative approaches. Short‐term but intensive xylogenesis monitoring allows for a precise definition of the cambial growing season and phenological phases. However, while this method offers relatively accurate insights into seasonal dynamics, it is less effective in capturing long‐term trends. In contrast, retrospective approaches—though less precise in determining the exact onset and end of the growing season—are most likely better suited to identifying long‐term changes in cambial phenology (Carrer et al. 2017). Further investigations are therefore needed to clarify whether the observed differences among regions and species truly reflect geographical and climatic distinctions, stem from species‐specific responses, or simply result from methodological inconsistencies between short‐ and long‐term observations or from model inaccuracies.

The relatively modest shift of cambial phenology compared to the faster onset recorded for leaf out aligns with the results presented by Lin et al. (2024) which estimated an earlier onset on cambial activity onset of 0.85–1.44 days per decade compared to around 1.26–1.94 days per decade for budburst.

Our observations are consistent with emerging evidence of a complex relationship between climate warming and wood formation dynamics with this latter lagging behind other phenological events. For instance, Huang et al. (2020) and Li et al. (2025) highlight that wood formation is predominantly driven by photoperiod and temperature, with secondary influences from spring forcing, winter chilling, and moisture availability. This means that while the accumulation of forcing temperatures can advance budburst, the onset of wood formation is less responsive to these changes. This dual effect contributes to the relatively smaller advancement of wood formation compared to other phenological events. Nonetheless, while some studies suggest that climate warming may increase chilling requirements and potentially delay phenological events due to reduced winter cold exposure, though likely more in warm biomes (Delpierre et al. 2019; Meng et al. 2021; Montgomery et al. 2020, Li et al., in press), our data still reveal a clear earlier onset of cambial activity. Even a shift of about 0.7 days per decade, as observed in our study, represents a meaningful change when considered over multiple decades, with potential implications for tree growth and related ecosystems carbon dynamics. Species and similar environmental settings appear to have a limited influence on the onset of wood formation in conifers across the Northern Hemisphere (Huang et al. 2020), as also supported by our study for the Eastern Alps, in which the differences between species and sites aren't statistically significant.

Our findings demonstrate that an indirect retrospective approach, when paired with detailed temperature records, can provide valuable long‐term insights into a hidden aspect of tree growth. We show that three of the most representative conifer species across the Alps exhibit similar rates of advance in the onset of cambial activity over the past two and a half centuries. These rates are significantly lower than those observed for other growth compartments (e.g., leaves, flowers) in the same region. Given that cambial activity plays a key role in forest carbon dynamics, as it is directly linked to wood formation, our results contribute to a better understanding of how trees respond to ongoing climate warming. If replicated across different regions and species, these findings could help improve the accuracy of global vegetation models.

## Author Contributions

**Eugenia Mantovani:** data curation, formal analysis, investigation, methodology, visualization, writing – original draft. **Angela Luisa Prendin:** data curation, formal analysis, methodology, supervision, visualization, writing – review and editing. **Michele Brunetti:** investigation, resources, writing – original draft, writing – review and editing. **Davide Frigo:** visualization, writing – review and editing. **Raffaella Dibona:** investigation. **Marco Carrer:** conceptualization, investigation, project administration, resources, supervision, writing – review and editing.

## Funding

This study was supported by NextGenerationEU, MSCA_0000018.

## Conflicts of Interest

The authors declare no conflicts of interest.

## Supporting information


**Data S1:** Supporting Information.

## Data Availability

The data that support the findings of this study are openly available in Zenodo at https://doi.org/10.5281/zenodo.17522058.
