# Supplementary material for: Phenological Shifts in Wood Formation Tracked by Frost Rings Across Two Centuries
Source: Glob Chang Biol. 2026 Feb 13;32(2):e70745. doi: 10.1111/gcb.70745 (PMC12902809; doi:10.1111/gcb.70745)

Supplementary material

Phenological Shifts in Wood Formation Tracked by Frost Rings Across Two Centuries

Eugenia Mantovani[1], Angela Luisa Prendin[1,2], Michele Brunetti[3], Davide Frigo[1], Raffaella Dibona[1], & Marco Carrer[1,3 *]

[1] Department of Land Environment Agriculture and Forestry (TeSAF), University of Padova, Legnaro, Italy.

[2] Section for Ecoinformatics and Biodiversity, Department of Biology, Aarhus University, Aarhus, Denmark.

[3] Institute of Atmospheric Sciences and Climate, National Research Council (CNR-ISAC), Bologna, Italy.

Figure S1: Climate of the studied regions

Figure S1: Climate conditions are summarized by Walter-Lieth climatographs, showing mean monthly precipitation (blue, mm), mean monthly temperature (red, °C), months in which frost events are likely to occur (light-blue boxes) and wet periods (dark-blue filled areas). Croda da Lago's (a) and Latemar's (b) (1850-2019).

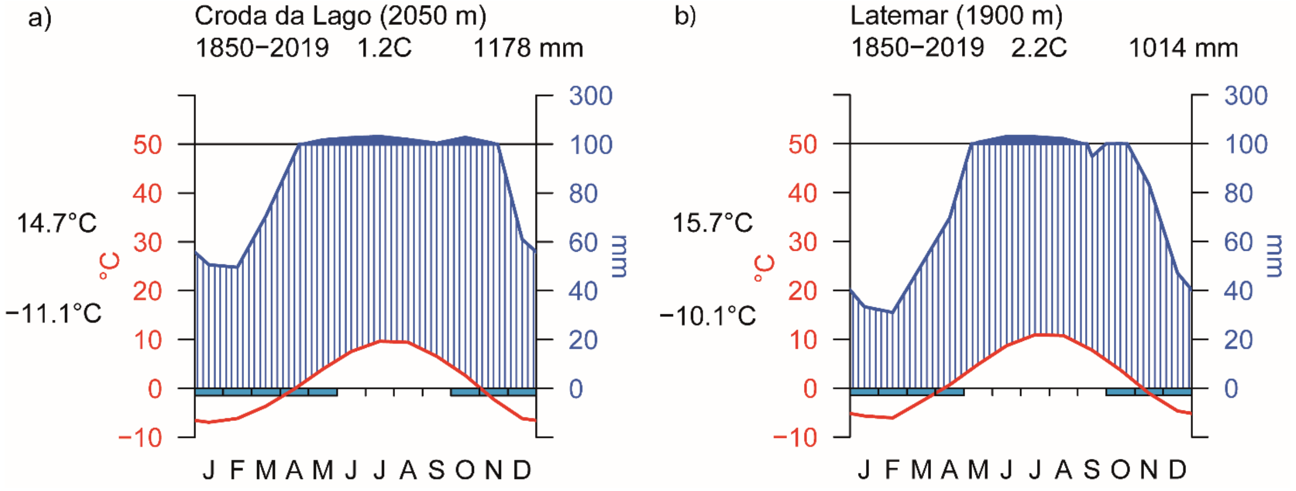

Table S1: Daily temperature reconstruction

Table S1: Summary of verification and error metrics for the April–June daily temperature reconstruction at Latemar and Croda da Lago, calculated over three sub-periods of equal length (statistics for anomalies are reported in parentheses). Values reported below the site names indicate the number of stations used for minimum and maximum temperature.

		Latemar		Croda da Lago		
		Tmin	Tmax	Tmin	Tmax	
	**N° of stations**	**4**	**4**	**4**	**4**	**Period 1774-1855**
Verification or Error metric	Pearson R	0.92 (0.80)	0.91 (0.83)	0.90 (0.79)	0.90 (0.83)	
	BIAS	0.36	0.36	0.67	0.67	
	MAE	2.11 (1.83)	2.11 (1.83)	2.30 (1.45)	2.30 (1.89)	
	RMSE	2.64 (2.36)	2.64 (2.36)	2.84 (1.85)	2.84 (2.40)	
	**N° of stations**	**55**	**54**	**52**	**51**	**Period 1856-1937**
Verification or Error metric	Pearson R	0.97 (0.91)	0.97 (0.92)	0.96 (0.90)	0.96 (0.92)	
	BIAS	0.22	0.78	0.37	1.13	
	MAE	1.27 (0.91)	1.61 (1.14)	1.33 (0.99)	1.84 (1.19)	
	RMSE	1.59 (1.22)	2.01 (1.56)	1.64 (1.30)	2.22 (1.57)	
	**N° of stations**	**1170**	**1178**	**1026**	**1034**	**Period 1938-2020**
Verification or Error metric	Pearson R	0.97 (0.94)	0.97 (0.95)	0.97 (0.95)	0.97 (0.96)	
	BIAS	0.12	0.65	0.26	1.01	
	MAE	1.14 (0.72)	1.46 (0.93)	1.12 (0.70)	1.61 (0.86)	
	RMSE	1.41 (0.98)	1.82 (1.30)	1.35 (0.93)	1.90 (1.16)	

MAE = mean absolute error; RMSE = Root Mean Square Error

Figure S2: Evaluation of the climate record reconstruction quality

Figure S2. Comparison of cold-spell reconstructions performed using all available station data (red; > 1,000 stations for both sites) and using only the stations available during the earliest period (1774–1855; black; 4 stations) for selected frost years occurring in recent decades. Additional reconstruction statistics are reported in Table S1.

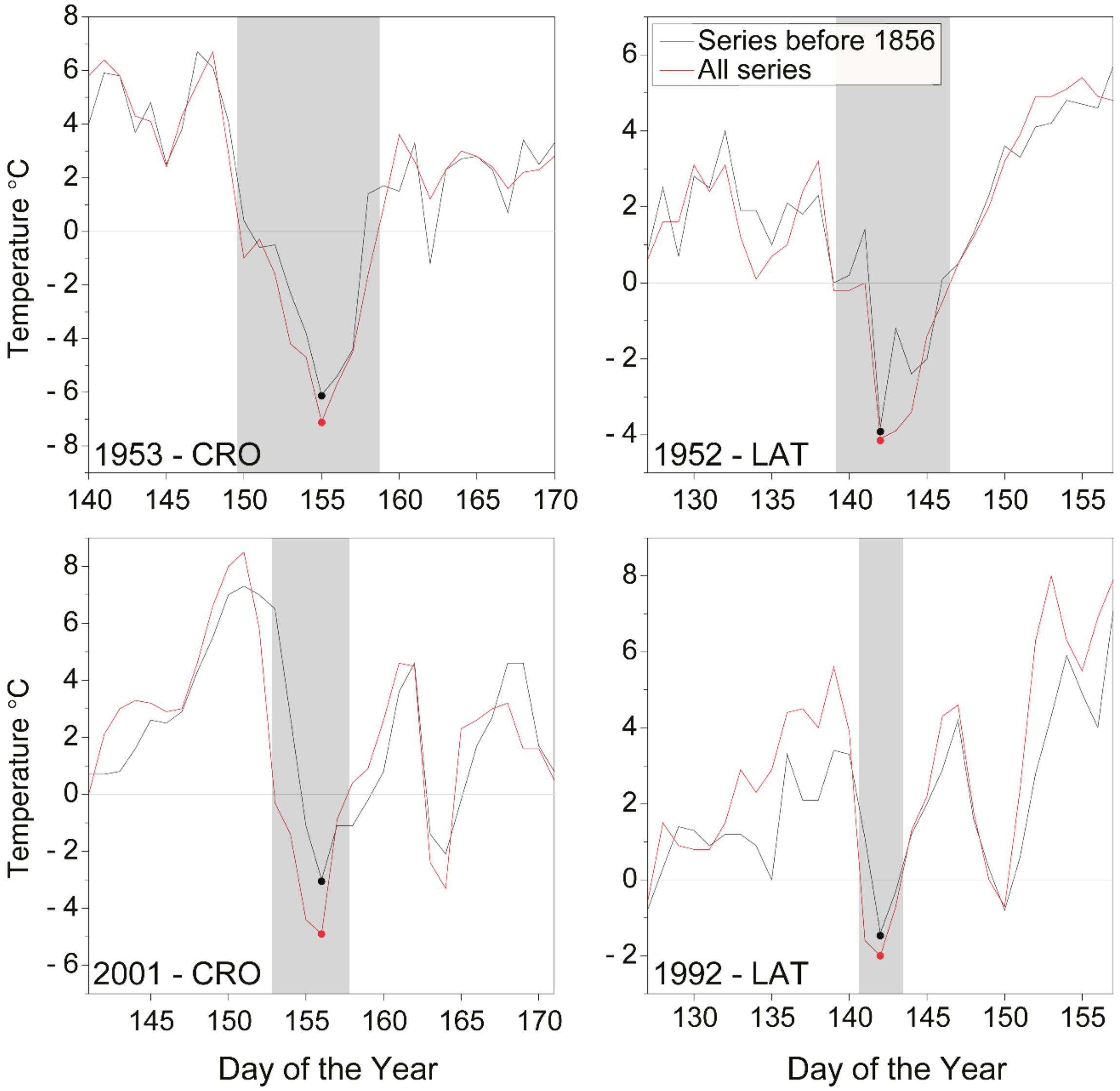

Table S2: Comparison of Linear, Exponential, and Power Models

Table S2: Comparison of R^2, Root Mean Squared Error (RMSE), and p-values for three models predicting frost Days: linear (Days ~ Year), exponential (log(Days) ~ Year), and power (log(Days) ~ log(Year)), considering the random factor (1 | Sp_Site). Results are reported overall, by species, by site, and by species × site combination. For groups including multiple Sp_Site levels, mixed-effects models with a random intercept for Sp_Site were fitted. For species × site subgroups containing only a single Sp_Site level, simple linear models were applied.

	Group	R^2 lin.	RMSE lin.	P-val lin.	R^2 exp.	RMSE exp.	P-val exp.	R^2 pow.	RMSE pow.	P-val pow.
**General**	All data	0.147	8.165	<0.001	0.147	8.167	<0.001	0.147	8.167	<0.001
**By Species**	*Picea abies*	0.215	7.846	<0.001	0.220	7.826	<0.001	0.222	7.811	<0.001
	Larix decidua	0.450	5.864	<0.001	0.443	5.894	<0.001	0.441	5.904	<0.001
	Pinus cembra	0.054	8.374	<0.001	0.054	8.377	<0.001	0.054	8.379	<0.001
**By Site**	Croda	0.208	7.447	<0.001	0.209	7.447	<0.001	0.208	7.449	<0.001
	Latemar	0.121	8.479	<0.001	0.121	8.482	<0.001	0.121	8.482	<0.001
**By Species-Site**	LADE_Croda	0.484	6.046	<0.001	0.478	6.086	<0.001	0.474	6.111	<0.001
	LADE_Latemar	0.389	4.989	0.012	0.383	5.016	0.012	0.382	5.023	0.012
	PIAB_Latemar	0.215	7.846	<0.001	0.220	7.826	<0.001	0.222	7.811	<0.001
	PICE_Croda	0.041	7.583	0.025	0.042	7.584	0.029	0.042	7.583	0.028
	PICE_Latemar	0.064	8.913	0.001	0.063	8.919	0.002	0.063	8.923	0.002

Table S3: Summary of Linear Mixed-Effects and Site-Specific Linear Models (accounting for frequency variability)

Table S3: Summary of model estimates and diagnostics for the linear mixed-effects model (LMM) and the site-specific linear model (LM). The LMM includes Year and Frequency as fixed effects and a random intercept for Sp_Site. Estimates are presented with standard errors (± SE) and degrees of freedom (df). Variance components for random effects (intercept) and residuals are reported along with marginal and conditional R^2 values. The LM includes site- and species-specific intercepts. R^2 and adjusted R^2 values are provided as overall model fit indices. Significance levels are denoted as: ***p < 0.001, **p < 0.01, * p< 0.05.

		**Estimate ± SE**	**DF**	**Cond. R^2 (Marg. R^2)**
**General (LMM)**	Intercept	288.08 ± 43.45***	89.99	0.23 (0.1)
	Year	-0.070 ± 0.023**	89.99	
	Frequency	-0.575 ± 19.96	87.82	
				**Mult. R^2 (Adj. R^2)**
**LADE_Latemar**	Intercept	519.76 ± 233.1	5	0.33 (0.19)
	Year	-0.197 ± 0.126		
**PICE_Latemar**	Intercept	294.70 ± 84.15**	26	0.1 (0.06)
	Year	-0.0736 ± 0.044		
**PIAB_Latemar**	Intercept	370.62 ± 87.65**	24	0.21 (0.18)
	Year	-0.117 ± 0.046*		
**LADE_Croda**	Intercept	335.93 ± 78.14**	12	0.29 (0.23)
	Year	-0.054 ± 0.093*		
**PICE_Croda**	Intercept	232.09 ± 90.25*	16	0.04 (-0.02)
	Year	-0.040 ± 0.047		

Figure S3: Assessment of temporal autocorrelation structures in the LMM

Figure S3: Autocorrelation function (ACF) plot of model residuals of the linear mixed effect model (LMM). The vertical bars represent autocorrelation values at different time lags, with the dashed blue lines indicating approximate 95% confidence limits.

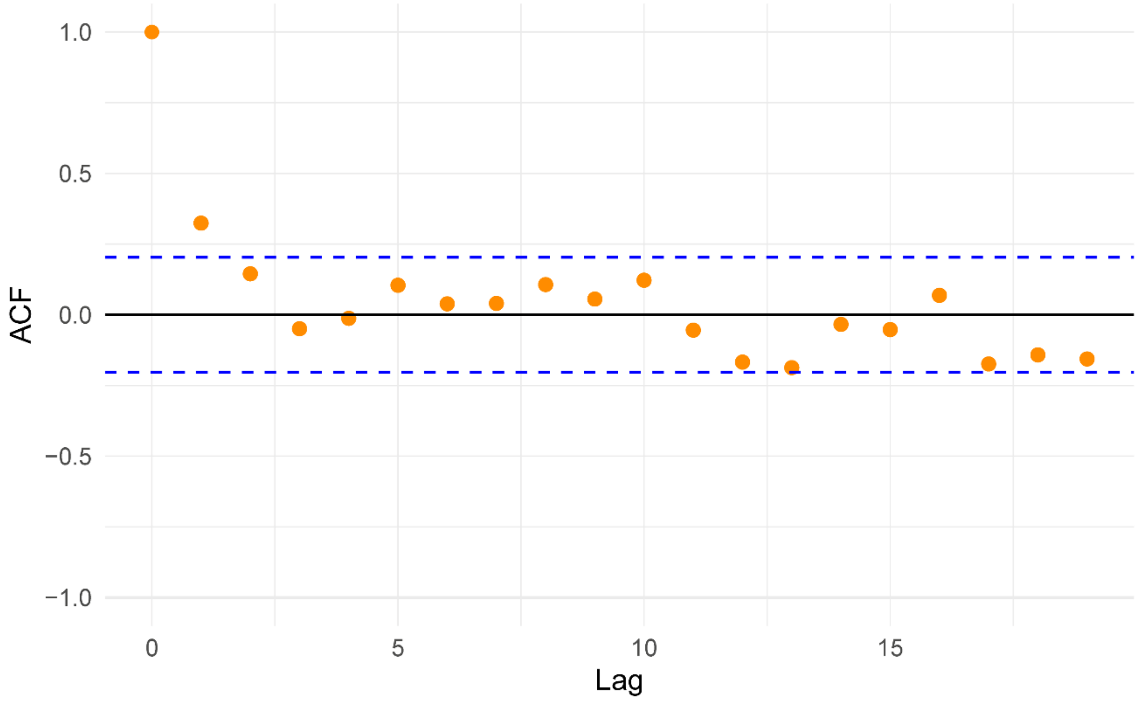

Table S4: Summary of models

Table S4: Summary of linear mixed-effects models (LMMs) evaluating the effects of Year, Frequency, Age (mean age of the trees that feature a frost ring in the same frost event), and their interactions on the response variable. Each model includes Species × Site (Sp_Site) as a random factor, with some models including an autoregressive correlation structure AR(1). Shown are the predictors included in each model, Akaike Information Criterion (AIC) values, parameter estimates ± standard errors (SE), and model fit statistics (marginal and conditional R^2). Marginal R^2 represents the variance explained by fixed effects, while conditional R^2 includes both fixed and random effects. Significance levels are denoted as: *** < 0.001, ** < 0.01, * < 0.05.

Models	Random/AutoCorr. Factor	AIC	Pred.	Estimate	Cond. R^2 (Marg. R^2)
Year + Frequency	(1 | Sp_Site)	724.72	Intercept	288.08 ± 43.45***	0.23(0.1)
			Year	-0.07 ± 0.023**	
			Frequency	-0.57 5± 19.96	
Year + Frequency	(1 | Sp_Site)+AR(1)	726.54	Intercept	286.178 ± 43.997***	0.23(0.09)
			Year	-0.069 ± 0.0230*	
			Frequency	-0.216 ± 20.665	
Year*Frequency	(1 | Sp_Site)	726.18	Intercept	289.64 ± 46.07***	0.22 (0.1)
			Year	-0.071 ± 0.024**	
			Frequency	-110.20 ± 966.73	
			Year:Frequency	0.059 ± 0.522	
Year* Frequency	(1 | Sp_Site)+AR(1)	727.9	Intercept	289.263 ± 46.661***	0.07(0.05)
			Year	-0.071 ± 0.025**	
			Frequency	-248.746 ± 990.475	
			Year:Frequency	0.134 ± 0.534	
Year + Frequency +Age	(1 | Sp_Site)	729.07	Intercept	285.39 ± 43.87***	0.23(0.1)
			Year	-0.068 ± 0.023**	
			Frequency	-1.499 ± 20.11	
			Age	-0.058 ± 0.107	
Year * Frequency + Age	(1 | Sp_Site)	730.52	Intercept	287.02±46.49**	0.22 (0.1)
			Year	-0.069 ± 0.024**	
			Frequency	-115.73 ± 970.85	
			Age	-0.058 ± 0.107	
			Year:Frequency	0.062 ± 0.524***	
Year * Frequency + Year*Age	(1 | Sp_Site)	742.18	Intercept	249.732 ± 66.680***	0.21(0.1)
			Year	-0.05 ± 0.035	
			Frequency	-28.642 ± 982.351	

			Age	3.481 ± 4.649	
			Year:Frequency	0.016 ± 0.53***	
			Year:Age	-0.002 ± 0.002	
Year + Age	(1 | Sp_Site)	734.9	Intercept	284.576 ± 41.605**	0.23(0.1)
			Year	-0.068 ± 0.022**	
			Age	-0.057 ± 0.106	
Year + Age	(1 | Sp_Site)+AR(1)	736.7	Intercept	282.748 ± 42.053***	0.23(0.1)
			Year	-0.067 ± 0.022*	
			Age	-0.066 ± 0.107	
Year * Age	(1 | Sp_Site)	746.7	Intercept	249.590 ± 59.912***	0.21 (0.1)
			Year	-0.0496 ± 0.0314	
			Age	3.473 ± 4.525	
			Year:Age	-0.002 ± 0.002	
Year * Age	(1 | Sp_Site)+ AR(1)	748.31	Intercept	244.856 ± 60.604***	0.07(0.05)
			Year	-0.047 ± 0.032	
			Age	3.713 ± 4.544	

Table S5: Model diagnostics

Table S5: Model diagnostics for the general linear mixed-effects model (LMM) and the site-specific linear model (LM). Metrics include corrected Akaike Information Criterion (AICc), Shapiro-Wilk test statistic (W) and corresponding p-value for assessing normality of residuals, and variance inflation factors (VIF) to evaluate multicollinearity among Year.

Model	AICc	Shapiro-Wilk (SW)	p-value SW	VIF
**LMM**	726.18	0.98	0.16	VIF(Year)=1.23
**LM**	729.35	0.98	0.14	VIF (Year)=2.3

Figure S4: Sensitivity analysis of age structure and day of frost definition

Figure S4: Sensitivity analysis (β ± 95% percentile confidence intervals) of the effect of Year (in blue) and the Julian day of frost (in orange). The “General” model includes all age data, while subsequent models sequentially exclude trees younger than 5 years, 10 years or specific 10-year age classes. “Bootstrap stratified” resamples data within each age class proportionally to their distribution in the reference year, preserving the original age structure. “Bootstrap unbalanced” applies inverse-probability weighting to account for unequal representation of age classes, assigning greater weight to underrepresented ones. Orange bars represent models evaluating uncertainty in the temporal definition of the frost day by shifting it by ±2 and ±3 days. The dashed vertical line indicates a β estimate of zero.

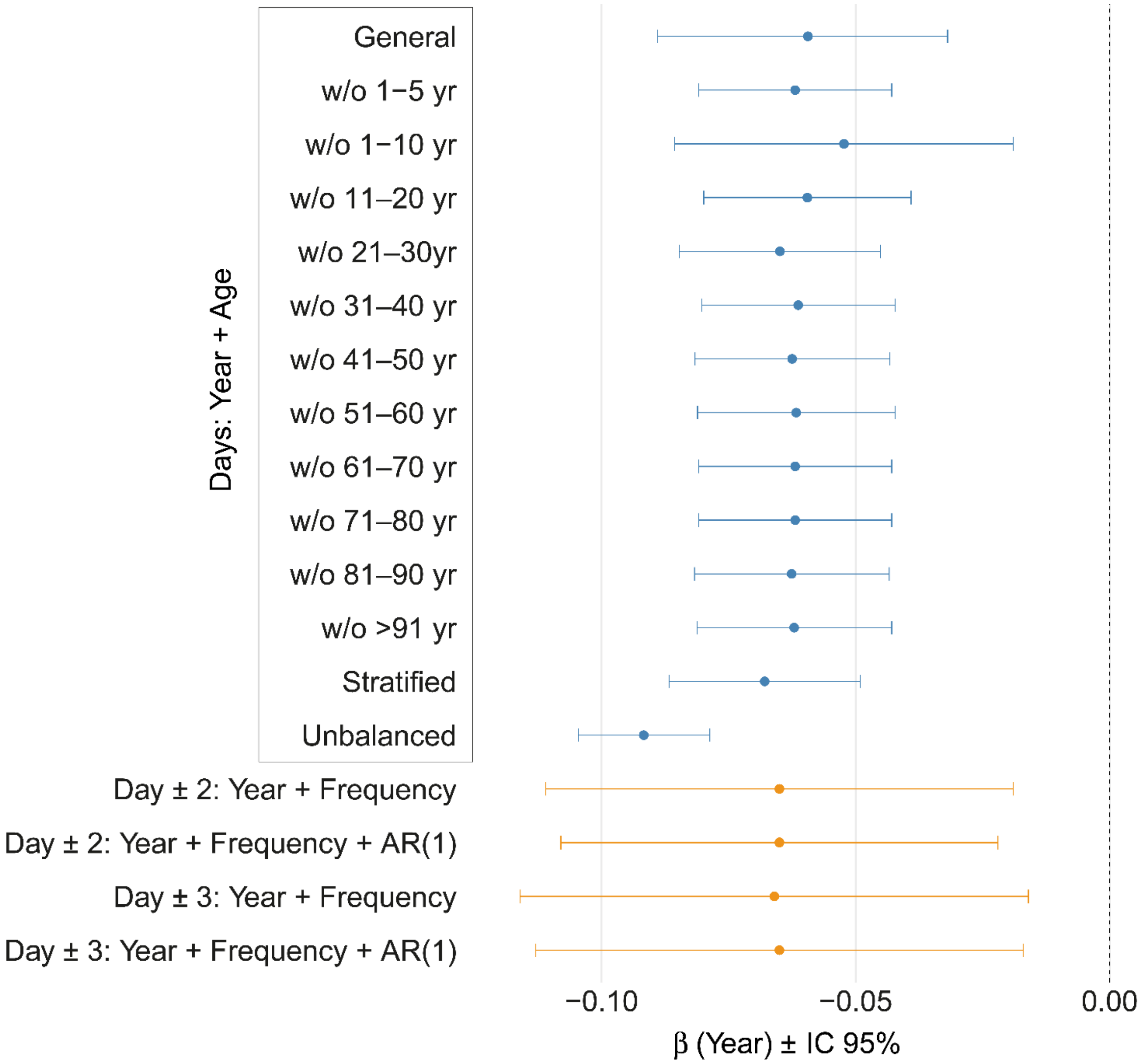

Table S6: Summary of Linear Mixed-Effects and Site-Specific Linear Models (accounting for age variability)

Table S6: Results of linear mixed-effects models (LMMs) and linear models (LM) assessing the effects of Year and Age on the response variable across different sites considering all the single trees (General, LADE_Latemar, PICE_Latemar, PIAB_Latemar, LADE_Croda, and PICE_Croda). Reported values are estimated coefficients ± standard errors (SE). Akaike's Information Criterion (AICc), conditional and marginal R^2 (Multiple and Adjusted R^2) values are provided for each model. Significance levels are denoted as: ***p < 0.001, **p < 0.01, * p< 0.05.

	AICc		Estimate ± SE	Cond. R^2 (Marg. R^2)
**General (LMM)**	3284.93	Intercept	272.176 ± 17.321***	0.29 (0.13)
		Year	-0.062 ± 0.009**	
		Age	-0.066 ± 0.042	
	3296.23	Intercept	296.200±21.980***	0.31 (0.14)
		Year	-0.074 ± 0.011***	
		Age	-2.831± 1.576	
		Year:Age	0.001±0.001	
				**Mult. R^2 (Adj. R^2)**
**LADE_Latemar**		Intercept	704.78 ± 217.291**	0.39 (0.28)
		Year	-0.297 ± 0.118*	
		Age	-0.020 ± 0.232	
**PICE_Latemar**		Intercept	230.910 ± 25.177***	0.07 (0.06)
		Year	-0.041 ± 0.013**	
		Age	-0.076± 0.091	
**PIAB_Latemar**		Intercept	380.271 ± 41.740***	0.22 (0.21)
		Year	-0.121 ± 0.023***	
		Age	-0.025 ± 0.062	
**LADE_Croda**		Intercept	389.541 ± 51.107***	0.49 (0.45)
		Year	-0.012 ± 0.026***	
		Age	0.077 ± 0.139	
**PICE_Croda**		Intercept	251.070 ± 42.873***	0.04 (0.03)
		Year	-0.05 ± 0.022*	
		Age	-0.039 ± 0.099	

Figure S5: Testing the legacy effect of the frost events.

Figure S5: Superposed Epoch Analysis (SEA) of tree-ring width individual series showing frost rings, standardized with a 20-year spline. The dark line indicates mean growth, while the shaded blue area represents the confidence intervals. The x-axis shows years relative to the frost event (year 0). The results are presented both at the site level and separately for each species within each site.

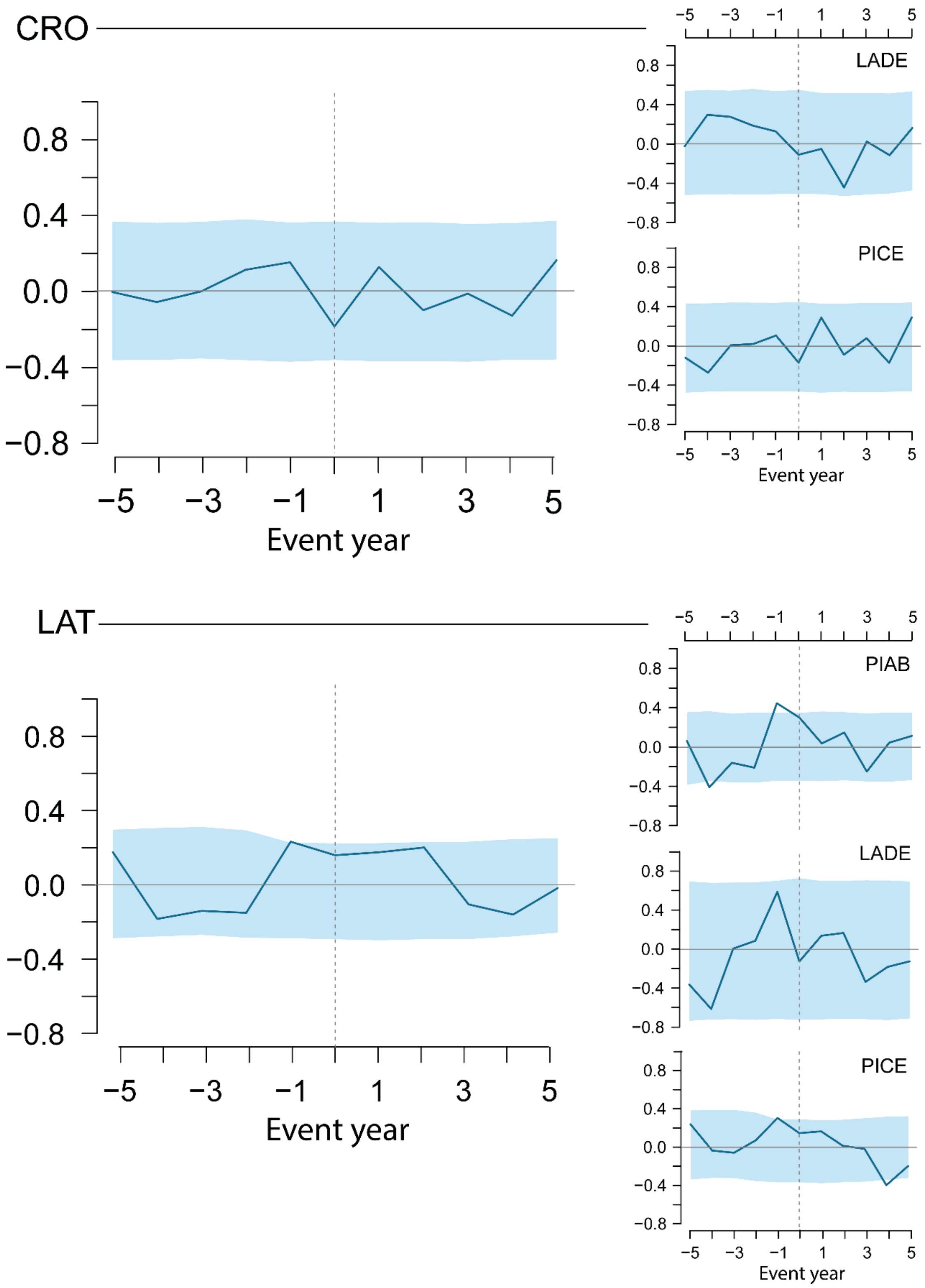

Supplement: Supplementary file 1 — Data S1: Supporting Information. [file GCB-32-e70745-s001.pdf]
